# Discussion on the recruitment strategy for apheresis platelet donors in Chongqing during a public health emergency^*^

**DOI:** 10.3389/fpubh.2024.1365433

**Published:** 2024-04-05

**Authors:** Ying Cheng, Chengbing Xie, Yunbo Tian, Fang Wang, Xingchen Liu, Danfeng Cheng

**Affiliations:** ^1^Chongqing Blood Center, Chongqing, China; ^2^School of Public Health, Chongqing Medical University, Chongqing, China

**Keywords:** public health emergency, apheresis platelets, characteristics, blood donors, recruitment strategy

## Abstract

**Objective:**

This study aimed to analyze the population characteristics of apheresis platelet donors in Chongqing Province and provide a scientific basis for the development of precise and efficient recruitment strategies. The ultimate goal is to increase the number of regular platelet donors in preparation for public health emergencies.

**Methods:**

This study involved 53,089 blood donors who donated apheresis platelets to the Chongqing Blood Center from 2020 to 2022. Data regarding age, sex, blood type, education level, occupation, and frequency of blood donation were collected and analyzed to identify factors influencing platelet donation.

**Results:**

Between 2020 and 2022, the majority of apheresis platelet donors in Chongqing were aged 25–35 years, with a male-to-female ratio of 2.6:1. The ABO blood group distribution was O > A > B > AB. The apheresis platelet donors mainly consisted of college students, and the donors who had donated only once accounted for the greatest proportion.

**Conclusion:**

Based on the population characteristics of apheresis platelet donors in Chongqing, blood collection and supply organizations must refine emergency blood collection and supply plans during public health emergencies. This study underscores the importance of developing precise and efficient recruitment strategies for apheresis platelet donors and expanding the pool of regular apheresis platelet donors. These measures are essential to ensure the timely, safe, and effective use of clinical blood resources during public health emergencies.

## 1 Introduction

With the advancing level of medical development in Chongqing and the implementation of universal health insurance policies, clinical component blood transfusions have become increasingly prevalent (1, 2). The clinical demand for apheresis platelets has increased accordingly (3, 4). However, due to the protracted collection process (5), specific storage requirements (6), and short shelf life of apheresis platelets (7), recruiting donors for this component has become a challenging endeavor, often resulting demand exceeding the available supply (8). Public health emergencies exacerbate this challenge and disrupt the balance between the supply and demand for apheresis platelets (9). The aim of this study was to investigate the recruitment strategies and effectiveness of apheresis platelets in recent years, aiming to reflect and summarize these strategies. By retrospectively analyzing the population characteristics of opportunistic blood donors in Chongqing from 2020 to 2022 and studying the factors associated with them, this study seeks to provide a scientific foundation for developing a set of precise recruitment strategies and retention measures, establishing a stable donor pool of opportunistic platelet donors, and effectively responding to public health emergencies.

## 2 Materials and methods

### 2.1 General materials

Data from 53,089 successful apheresis platelet donors at the Chongqing Blood Center between January 1, 2020, and December 31, 2022 were collected. All donors met the health examination requirements for blood donation. The study subjects included 38,346 males and 14,743 females. The 25–35 age group had the most donors, comprising 16,904 individuals (31.8%), with the highest proportion of first-time donors (53.6%).

### 2.2 Methods

Blood donor data were extracted from the BMIS system of the Chongqing Blood Center. Relevant information concerning platelet donation from apheresis platelet donors was categorized and compared based on sex, age, blood type, education level, and blood donation frequency. The correlation between these population characteristics and platelet donation was analyzed.

### 2.3 Statistical analysis

We used Excel and SPSS 26.0 statistical software for data analysis. Count data were presented as rates or constituent ratios, and the *χ*^2^ test was used. A significance level of *p* < 0.05 was considered to indicate statistical significance.

## 3 Results

### 3.1 Age and sex distribution of the blood donors of apheresis-treated platelets

Between 2020 and 2022, the majority of donors of apheresis platelets were aged 25 to under 35 years, accounting for 31.8%. The second-largest age group was 18–25 years, representing 28.1% of the donors. In terms of gender distribution, male donors comprised 72.2% of the donors, while female donors constituted 27.8%, resulting in a male-to-female ratio of 2.6:1 (Table 1).

**Table 1:** Age and gender distribution of organically apheresis platelets donors, 2020–2022.

Year	Number of blood donors	Age					Gender	
		18–25	25–35	35–45	45–55	55–60	Male	Female
2020	14,918	3,737 (25.1)	5,105 (34.2)	2,869 (19.2)	2,711 (18.1)	496 (3.3)	10,814 (72.5)	4,104 (27.5)
2021	18,888	5,944 (31.5)	5,656 (29.9)	3,250 (17.2)	3,326 (17.6)	712 (3.8)	13,592 (72.0)	5,296 (28.0)
2022	19,283	5,218 (27.1)	6,143 (31.9)	3,504 (18.2)	3,600 (18.7)	818 (4.2)	13,740 (71.3)	5,543 (28.7)
Total	53,089	14,899 (28.1)	16,904 (31.8)	9,623 (18.1)	9,637 (18.2)	2026 (3.8)	38,146 (71.9)	14,943 (28.1)

Comparisons of different age groups of blood donors in different years, *χ*^2^ = 224.205, *p* < 0.01; *χ*^2^ = 6.514, *p* < 0.05 for sex comparisons.

### 3.2 Distribution of blood types of apheresis platelet donors

The percentage distribution of ABO blood types among machine-recovered platelet donors from 2020 to 2022 was as follows: 32% A, 24% B, 34% O, and 10% AB. In descending order, the order was O > A > B > AB (Table 2).

**Table 2:** Distribution of ABO blood groups among machine-recovered platelet donors during 2020–2022.

Year	Number of blood donors	A	B	O	AB
2020	14,918	4,826 (32.4)	3,469 (23.3)	5,090 (34.1)	1,533 (10.3)
2021	18,888	5,852 (31.0)	4,472 (23.7)	6,418 (34.0)	2,146 (11.4)
2022	19,283	6,195 (32.1)	4,606 (23.9)	6,517 (33.8)	1965 (10.2)
Total	53,089	16,873 (31.8)	12,547 (23.6)	18,025 (34.0)	5,644 (10.6)

Comparison of the distribution of blood groups among donors in different years, *χ*^2^ = 22.520, *p* < 0.01.

### 3.3 Distribution of the literacy level of machine-recovered blood donors

The general education level of machine-recovered platelet donors in 2020–2022 was relatively low. However, the number of donors with at least a high school education or higher was on the rise each year (Table 3).

**Table 3:** Distribution of literacy levels of donors during 2020–2022.

Year	Number of blood donors	Junior high school and below	Senior high school	Secondary technical school	Junior college	Undergraduate	Postgraduate and above	Other
2020	14,918	3,579 (24.0)	3,798 (25.5)	455 (3.1)	3,099 (20.8)	2,657 (17.8)	269 (1.8)	1,061 (7.1)
2021	18,888	5,407 (28.6)	4,047 (21.4)	401 (2.1)	3,987 (21.1)	3,671 (19.4)	302 (1.6)	1,073 (5.7)
2022	19,283	3,820 (19.8)	4,612 (23.9)	388 (2.0)	4,435 (23.0)	3,806 (19.7)	517 (2.7)	1705 (8.8)
Total	53,089	12,806 (24.1)	12,457 (23.5)	1,244 (2.3)	11,521 (21.7)	10,134 (19.1)	1,088 (2.0)	3,839 (7.2)

Comparison of the literacy levels of blood donors in different years, *χ*^2^ = 647.859, *p* < 0.01.

### 3.4 Occupational distribution of machine blood donors

Between 2020 and 2022, the primary group of machine blood donors consisted of university students (22%), followed by employees at 12% (Table 4).

**Table 4:** Occupational distribution of blood donors during 2020–2022.

Year	Number of blood donors	Other	Student	Liberal professions	Office clerk	Peasant	Business service personnel	Medical staff	Civil servant
2020	12,800	5,814 (45.4)	2,183 (17.1)	1,648 (12.9)	1,501 (11.7)	827 (6.5)	351 (0.03)	274 (2.7)	202 (2.1)
2021	16,449	7,058 (42.9)	4,198 (25.5)	1,607 (9.8)	1,695 (10.3)	932 (5.7)	386 (2.3)	341 (2.1)	232 (1.4)
2022	16,256	7,325 (45.1)	3,677 (22.6)	1,381 (8.5)	2,136 (13.1)	872 (5.4)	304 (1.9)	319 (2.0)	242 (1.5)
Total	45,505	20,197 (44.4)	10,058 (22.1)	4,636 (10.2)	5,332 (11.7)	2,631 (5.8)	1,041 (2.3)	934 (2.1)	676 (1.5)

Comparison of the occupations of blood donors in different years, *χ*^2^ = 486.925, *p* < 0.01.

### 3.5 Frequency of blood donation and percentage of frequency in the population of apheresis platelets

The highest proportion of apheresis platelet donors in 2020–2022 donated only once, accounting for 53.6% of the total donors. In contrast, the next largest group of donors was those who donated twice (15.2%), as indicated in Table 5.

**Table 5:** Frequency and percentage of frequency of blood donations in the apheresis platelets population during 2020–2022.

Year	Number of blood donors	1	2	3	4	5	6	7	8	≥9
2020	5,541	3,488 (62.9)	685 (12.4)	335 (6.0)	222 (4.0)	128 (2.3)	117 (2.1)	79 (1.4)	81 (1.5)	406 (7.3)
2021	5,604	2,758 (49.2)	953 (17.0)	447 (8.0)	272 (4.9)	191 (3.4)	156 (2.8)	105 (1.9)	98 (1.7)	624 (11.1)
2022	5,691	2,779 (48.8)	916 (16.1)	461 (8.1)	299 (5.3)	209 (3.7)	170 (3.0)	126 (2.2)	104 (1.8)	627 (11.0)
Total	16,836	9,025 (53.6)	2,554 (15.2)	1,243 (7.4)	793 (4.7)	528 (3.1)	443 (2.6)	310 (1.8)	283 (1.7)	1,657 (9.8)

Comparison of the frequency of blood donation among donors in different years, *χ*^2^ = 301.366, *p* < 0.01.

### 3.6 Analysis of the number of apheresis platelet donors and person-times

The percentages of first-time donors in 2020, 2021, and 2022 were 37.92, 23.95, and 33.84%, respectively, as depicted in Table 6.

**Table 6:** Analysis of the number of organically apheresis platelets donors, person-times during 2020–2022.

Year	Number of initial mechanogenic platelet donors	Total number of mechanogenic platelet donors for the year	initial donors/total donors (%)	Total blood donations during the year	Average number of blood donations per person during the year
2020	2,101	5,541	37.92	14,918	2.7
2021	1,342	5,604	23.95	18,888	3.37
2022	1926	5,691	33.84	19,283	3.39

## 4 Discussion

With the increasing level of healthcare provision in Chongqing in recent years, there has been a consistent year-on-year increase in the demand for clinical platelets. During the period from November to December 2022, apheresis platelet collection in Chongqing reached an unprecedented low point in terms of availability (7). In response, the Chongqing Blood Center intensified its efforts by increasing platelet recruitment through various means, including using news media, advertising, telephone outreach, and the micro-letter platform. These measures were aimed at disseminating timely information regarding clinical platelet demand and assembling a comprehensive apheresis platelet emergency blood donation team comprising experts, leaders, cadres, and blood donors. The primary objective was to enhance apheresis platelet collection to ensure the fulfillment of clinical blood demand (10).

The survey findings revealed that the age group with the highest proportion of apheresis platelet unremunerated blood donors in Chongqing was individuals aged 25 to younger than 35 years, constituting 32% of the donors (Table 1). This pattern was consistent with relevant reports (5). Most of these young donors were in good health, with a strong commitment to blood donation and a significant sense of social responsibility. Furthermore, these donors generally possess substantial knowledge about the blood donation process, both before and after donation. Consequently, these individuals experience less psychological stress and fewer adverse reactions during donation, aligning with reports from other regions across the country (6). Notably, the male-to-female ratio among single platelet unpaid blood donors in Zhengzhou is 2.6:1 (Table 1), which is similar to the sex distribution in Zhengzhou (7) but differs from that in other parts of China (11). The underrepresentation of female donors can be attributed to various factors, including physiological reasons such as menstruation, pregnancy, and lactation, as well as psychological factors such as a fear of anemia, needle-related anxiety, apprehension about disease transmission, and general blood donation anxiety. This greater proportion of male donors than of female donors results in an imbalance in the sex ratio of single-recipient platelet donors (12). To address this issue, it is essential to understand the primary motivations and obstacles specific to female blood donation and devise tailored strategies to encourage female participation. Promoting blood donation awareness among women is crucial, as it emphasizes the importance of blood donation for the well-being and health of the population and ensures that women are well informed about the safety of the process, thereby improving their motivation to donate blood.

The findings of this study indicated that the distribution of blood types among apheresis platelet donors in this city was as follows: type A, 32%; type B, 24%; type O, 34%; AB, 10%; and O > A > B > AB (Table 2). This trend has remained consistent over the course of 3 years, differing from certain regions in China but aligning with the blood type distribution observed in most other parts of China. The reasons for this inconsistency in our study can be attributed primarily to geographical variations. Distinct geographic locations and climate conditions in different areas can influence the prevalence of specific blood types among donors. For instance, certain regions may have a greater propensity for specific blood types due to climate, topography, and other environmental factors. Furthermore, the economic development of different regions may also impact the distribution of blood types among donors (13). Regions with more rapid economic growth tend to attract donors with specific blood types, owing to the availability of medical resources and effective blood donation promotion. Cultural differences and traditions across regions can also play a role in shaping the distribution of blood types. Some areas may pay more attention to donors of particular blood types, while cultural backgrounds in other regions may influence donor choices. The location and distribution of blood donation centers in various regions can further affect the proportion of blood types in the donor pool. In some areas, blood donation sites may be concentrated in specific regions, leading to variations in donor proportions compared to those in other areas. Population mobility and migration within different regions may also lead to changes in the distribution of blood groups among donors. High levels of population movement and migration in certain areas can result in shifts in the proportion of blood types among donors in those regions.

The educational background of apheresis platelet donors predominantly consisted of individuals with a high school education or below in the first 2 years. However, there was an improvement in 2022, with an increase in the number of donors possessing specialized education and an uptick in donors with postgraduate education (Table 4). This suggests that as society progresses, the cultural and educational levels of the population are increasing. Individuals with higher education levels exhibit greater awareness and appreciation of NRL. For this specific segment of the population, several actions should be taken. First, there should be increased public awareness efforts to encourage blood donors to become advocates of blood donation. Second, enhanced services, including thorough pre-donation preparations, a comfortable blood donation environment, and the presence of professional medical staff, should be provided to these donors. This approach is aimed at ensuring that donors feel valued and cared for in their altruistic blood donation endeavors. Additionally, increasing the number of blood donation centers in regions with a concentration of highly educated individuals would enhance accessibility and make it more convenient for them to participate in voluntary blood donation. Efforts should also be directed toward encouraging apheresis platelet donors who have already participated in non-remunerative blood donation to continue their contributions. This can be achieved by offering relevant guidance and support, such as nutritional advice and health check-ups.

Because there are the numerous occupational categories, the top eight occupations were listed individually, while the remaining occupations, categorized as “others,” accounted for the greatest proportion at 44%. This situation arouse from the fact that certain professions were not explicitly categorized in the blood donation registration, leading individuals to select “other occupations,” making it challenging to determine their specific lines of work. Additionally, repeat donors no longer reiterated their occupations in subsequent blood donation registrations, as only the most recent occupation was documented. Some individuals also opted to not disclose their occupation, citing privacy concerns or other personal reasons.

Blood donors can be found across all sectors of society, but the percentage of blood donors varies among different occupations. When breaking down these occupations, the greatest proportion of blood donations primarily come from individuals categorized as “other people.” This broad category included freelancers, non-engaged workers, and industrial/commercial self-employed individuals, among others. The majority of individuals in this group had the freedom to manage their time and were not constrained by specific time or location limitations in regard to platelet donation. Consequently, this group contributed significantly to the overall blood donation pool. Public officials (10), on the other hand, were characterized by their sense of righteousness and altruism, good health, and a strong initiative to participate in blood donation. On the contrary, teachers, national civil servants, and medical personnel exhibited relatively lower rates of blood donation. This was likely due to the inconvenience of blood donation center locations and time constraints, particularly for medical personnel who often worked irregular shifts. Additionally, machine-recovered platelet donations took longer than whole blood donations, contributing to reduced participation from this segment of the population (14–16).

College students played a pivotal role in blood donation, accounting for 22% of the total donations. They constituted an essential group of non-remunerated blood donors and a reliable source of safe component blood donation. College students generally exhibited a high level of cultural sophistication, were open to new experiences, maintained good physical health, and had a high success rate in blood testing. Their motivation to donate blood was characterized by a clear sense of honor and strong social responsibility. The presence of numerous colleges and universities in the Chongqing region, coupled with the flexibility of college students’ class schedules, made apheresis platelet donation more convenient for them compared to the general community. This convenience factor contributes to the greater proportion of apheresis platelet donations among college students (17). Furthermore, the diversity of our blood donation recruitment activities within colleges and universities, along with ongoing improvements in publicity efforts, has resulted in an increasing acceptance of blood donation among college students. In the future, efforts should focus on tapping into the potential of college students, intensifying blood donation promotion and recruitment in educational institutions, and encouraging low-risk unpaid blood donors to join machine-recovered platelet teams, thereby establishing a strong foundation for the retention of apheresis platelet donors (18).

Regarding the frequency of blood donation, the primary proportion of donors who made a single donation in 2020 was 23%. In response to this status quo, the center implemented a series of retention policies. Although the proportion of first-time donors decreased significantly in 2021 and 2022, the percentage of individuals who donated only once remained the highest. This underscores the need to enhance recruitment strategies for new donors while also focusing on retaining existing donors. In 2020, a total of 14,918 individuals engaged in apheresis platelet donation, while 5,541 people participated in platelet donation. On average, each person made 2.69 donations. In 2021, 1,888 individuals participated in apheresis platelet donation, and 5,604 individuals participated in platelet donation, with an average of 3.37 donations per person. In 2022, 19,283 individuals participated in apheresis platelet donation, and 5,691 individuals participated in platelet donation, with an average of 3.37 donations per person. The number of platelet donations per person in 2022 was 3.39 on average, showing stability compared to the 3.37 donations per person in 2021. This was achieved while maintaining a relatively high level of donation frequency during the most critical phase of the COVID-19 pandemic. As we transition into the post-pandemic era, it becomes increasingly vital to employ diverse methods for promoting voluntary blood donation. We should offer personalized incentives to boost the motivation of blood donors, further enhance apheresis platelet donation frequency (19), streamline the blood donation service process, create a more comfortable environment for blood donation, elevate donor satisfaction, and continue dedicated efforts to retain donors and expand the pool of regular unpaid blood donors. These measures are essential to ensure a consistent and adequate blood supply for clinical use.

Considering the demographic characteristics of the center’s organic blood donors and the specific conditions in Chongqing, the center has devised a series of significant strategies and outlined various recruitment and donor retention strategies for Golden Ideas over the past 3 years: (1) strengthening the promotion of voluntary blood donation: Utilizing the center’s non-remunerated blood donation science and health education museum, staff members explained the knowledge related to organic platelet donation, blood utilization policies, the blood donation process, and pre, during, and post-donation precautions. This approach aims to alleviate donor concerns, enhance their willingness to donate blood, and boost their motivation to do so, especially among female donors and individuals with relatively higher literacy levels. (2) Partner program: given the low rate of repeated organic platelet donations in the Chongqing region, a program is planned to have established donors guide new donors in blood donation. Upon successful blood donation, new donors receive incentives. This program combines the number of successful blood donations with the number of blood donation souvenirs. It serves to encourage the enthusiasm of existing donors and expand the pool of organic platelet donors. (3) Expanding the fleet of mobile blood donation vehicles for organic platelet collection: This facilitates blood donation by public servants, educators, and individuals in the commercial service sector, increasing the proportion of blood donations from these occupational groups. (4) Face-to-face role models: gold medal recipients in blood donation provide insights and experiences on blood donation to other donors on-site. Their testimonials encourage more individuals to donate blood and expand the group of apheresis platelet blood donors. (5) A reserve pool of platelet donors, based on different blood types, should be established, and targeted recruitment efforts, according to specific blood types, also should be carried out to meet clinical requirements. (6) Regular networking activities should be implemented to enhance communication and interaction with blood donors, thereby increasing the frequency of blood donation. (7) Providing birthday cakes and cards to donors on their birthdays to maintain unpaid regular blood donors and enhance the percentage of blood donors from specific professions. (8) Introducing the “Neighborhood Partnering” mode, allowing regular OPC donors to promote blood donation in their communities to recruit additional stable, low-risk OPC donors.

In conclusion, during public health emergencies, it is imperative to analyze the characteristics of the unpaid blood donation population, consider the local context, and adapt recruitment strategies for apheresis platelets. These strategies should be continuously refined and tailored to efficiently expand the pool of unpaid machine-recovered platelet blood donors. This proactive approach is essential to address potential challenges such as donor losses and increased demand (20). Additionally, enhancing communication and engagement with the public and improving professional and customer-oriented approaches in blood donation services are vital to attract more individuals to participate in blood donation. These efforts are crucial to meet future challenges in the field of machine-recovered platelets in Chongqing and ensure the fulfillment of clinical requirements for apheresis platelets.

## Data availability statement

The raw data supporting the conclusions of this article will be made available by the authors, without undue reservation.

## Author contributions

YC: Funding acquisition, Writing – original draft, Writing – review & editing. CX: Methodology, Project administration, Resources, Writing – review & editing. YT: Conceptualization, Software, Supervision, Writing – review & editing. FW: Writing – review & editing, Validation, Visualization. XL: Writing – original draft, Data curation, Formal analysis, Investigation. DC: Funding acquisition, Writing – original draft, Writing – review & editing. All authors read and approved the final manuscript.
